# The cost-effectiveness of celecoxib versus non-steroidal anti-inflammatory drugs plus proton-pump inhibitors in the treatment of osteoarthritis in Saudi Arabia

**DOI:** 10.1186/s13561-015-0053-7

**Published:** 2015-06-11

**Authors:** Sherif A Nasef, A. Aziz Shaaban, Joaquin Mould-Quevedo, Tarek A Ismail

**Affiliations:** King Fahd Hospital-Dammam, 6830 Ammar Bin Thabit St, Al Muraikabat, Dammam, 32253-3202 Kingdom of Saudi Arabia

**Keywords:** Celecoxib, Non-steroidal anti-inflammatory drugs, Saudi Arabia, Cost effectiveness, Osteoarthritis

## Abstract

**Background:**

Cyclooxygenase (COX)-2 inhibitors including celecoxib are as effective as non-selective non-steroidal anti-inflammatory drugs (ns-NSAIDs) in the treatment of osteoarthritis (OA) and have less gastrointestinal toxicity. Although they are associated with higher treatment costs, COX-2 inhibitors may simultaneously reduce costs associated with adverse events, hence, their overall economic benefit should be assessed.

**Objective:**

To evaluate the incremental cost effectiveness ratio (ICER) of celecoxib versus ns-NSAIDs, with/without proton-pump inhibitor (PPI) co-therapy, for managing OA in Saudi Arabian subjects aged ≥65 years.

**Methods:**

The National Institute for Health and Care Excellence health economic model from the UK, updated with relative risks of adverse events using CONDOR trial data, was adapted. Patients received celecoxib or ns-NSAIDs, with/without omeprazole. The effectiveness measure was quality-adjusted life years (QALYs) gained per patient. The analysis was conducted from the patient’s perspective. Frequencies of resource use for adverse events were based on data collected in July 2012 from seven private hospitals in Jeddah, Saudi Arabia. Probabilistic sensitivity analysis was performed to construct cost-effectiveness acceptability curves (CEACs).

**Results:**

Over a 6-month treatment duration, QALYs gained per patient were higher with celecoxib (0.37) and celecoxib plus PPI (0.40) versus comparators. Ibuprofen plus PPI showed the lowest expected cost per patient (US$ 1,314.50 versus US$ 1,422.80 with celecoxib plus PPI and US$ 1,543.50 with celecoxib). Celecoxib plus PPI was the most cost-effective option with an ICER of US$ 1,805.00, followed by celecoxib (ICER, US$ 7,633.33) versus ibuprofen plus PPI. Over 2- and 5-year treatment durations, celecoxib plus PPI, and celecoxib, showed higher QALYs gained/patient and lower ICERs versus comparators. These ICERs are <1 gross domestic product/capita in Saudi Arabia in 2013 (US$ 25,961).

CEACs over 6 months’ treatment showed a significantly higher likelihood that celecoxib plus PPI and celecoxib alone would be more cost effective versus comparators once the willingness to pay is over US$ 2,000.00.

**Conclusion:**

After considering new adverse event risks, celecoxib with/without PPI co-therapy was deemed very cost effective for medium- and long-term use in Saudi Arabian OA patients aged ≥65 years.

## Background

Osteoarthritis (OA) is a widely prevalent condition, which is associated with significant morbidity and quality-of-life issues. According to the World Health Organization (WHO), up to 40 % of the population aged >70 years suffers from OA, with 80 % of patients having some form of movement limitation and 25 % having problems in performing their daily activities [1].

Studies show that pain is an important cause of disability and reduced function in OA patients [2, 3]. Paracetamol and non-steroidal anti-inflammatory drugs (NSAIDs) are the mainstay of pharmacological management for controlling pain and stiffness in OA [4]. Oral NSAIDs are recommended in patients who do not respond to full-dose paracetamol. However, NSAIDs are associated with adverse effects on the gastrointestinal (GI), cardiovascular (CV) and renal systems. Furthermore, the risk of GI bleeding increases with age, and in subjects with stomach ulcers and bleeding problems [5]. Some cyclooxygenase (COX)-2 inhibitors such as celecoxib are associated with less GI and CV adverse reactions than non-selective NSAIDs (ns-NSAIDs) [5].

To reduce the risk of GI toxicity, the 2008 National Institute for Health and Care Excellence (NICE) Clinical Guideline for managing OA in adults recommends co-prescribing both selective and ns-NSAIDs with proton-pump inhibitors (PPIs) [5]. Celecoxib versus Omeprazole aNd Diclofenac in patients with Osteoarthritis and Rheumatoid arthritis (CONDOR) was a landmark trial that was published after the NICE Clinical Guideline. It showed that the proportion of patients developing clinically significant GI events throughout the GI tract was significantly lower among those receiving celecoxib versus the ns-NSAID diclofenac plus omeprazole (hazard ratio [HR], 4.3 in favour of celecoxib; 95 % confidence interval [CI], 2.6 to 7.0; p < 0.0001) [6].

According to the NICE Clinical Guideline for managing OA in adults, the choice of NSAID and PPI should be determined by the cost of these agents [5]. A health economic model developed by NICE demonstrated that treatment with celecoxib plus PPI was more cost-effective than that with diclofenac plus PPI [5]. Furthermore, it has been shown that it is cost effective to co-prescribe a PPI with a COX-2 inhibitor even in patients who have low risk of GI adverse events [5, 7].

There are sparse data on the epidemiology of OA in Saudi Arabia. A study conducted in 1995 among 5,894 Saudi Arabian adults found OA in 13 % of the subjects. In line with global data, the prevalence of OA was high in the elderly population (30.8 % in those aged 46–55 years and 60.6 % in those aged 66–75 years) [8]. Another recent study found that elderly Saudi patients with OA had significantly higher pain scores and physical disability than those without OA, leading to a negative impact on their quality of life [9]. Clearly, there is an important role for pain medications in the management of OA in Saudi Arabia.

The Saudi Ministry of Health is the major government provider and financer of healthcare services in Saudi Arabia, responsible for 60 % of the total health services in the country; other government bodies, including specialist referral hospitals, security forces and army medical services, and school health units of the Ministry of Education, account for another 20 % [10]. Private sector healthcare services make up the remaining 20 %, important particularly in cities and large towns. As services in government facilities are provided free-of-charge, a considerable cost pressure is exerted on the national government [10]. This prompted the establishment of a cooperative health insurance scheme in 1999. Cooperative health insurance is applicable to Saudi citizens and expatriates in the private sector, in which employers are mandated to pay for employees’ health coverage costs [10]. From one insurance company in 2004, the providers of cooperative health insurance now number 25 companies.

Data on the cost effectiveness of drugs for OA will be invaluable to health policy makers, insurance providers and healthcare professionals, allowing better informed decision-making when choosing treatments to fund or utilize.

The objective of this study was to determine the cost effectiveness of celecoxib versus ns-NSAIDs, with and without PPI co-therapy, for managing OA in subjects aged ≥65 years in Saudi Arabia.

## Methods

This cost effectiveness analysis (CEA) was performed according to the recommendations described by NICE [11]. The primary outcome of cost effectiveness was quality-adjusted life years (QALYs). A patient perspective was adopted for the analysis as cost of treatment is borne by patients in Saudi Arabia.

### Comparators

Data from five large randomized controlled trials that report GI and CV events associated with NSAID use in predominantly OA patients were used to obtain adverse event data for this analysis. These included Celecoxib Long-term Arthritis Safety Study (CLASS; celecoxib, ibuprofen and diclofenac) [12], the Multinational Etoricoxib and Diclofenac Arthritis Long-term study (MEDAL; etoricoxib and diclofenac) [13], the Therapeutic Arthritis Research and Gastrointestinal Event Trial (TARGET; lumiracoxib, naproxen and ibuprofen) [14, 15], the Etoricoxib versus Diclofenac Sodium Gastrointestinal Tolerability and Effectiveness trial (EDGE; etoricoxib and diclofenac) [16] and CONDOR [6]. Of these, CLASS and MEDAL also included some patients with rheumatoid arthritis (RA). However, the relative risk of adverse events was considered to be similar in subjects with OA and RA as there is no evidence to suggest that rates of drug-induced adverse events are related to the type of arthritis [7]. These studies permit comparison between COX-2 inhibitors (celecoxib and etoricoxib) and ns-NSAIDs (diclofenac, ibuprofen and naproxen). In this CEA, we compared the cost effectiveness of celecoxib (200 mg OD) versus ibuprofen (400 mg TID), diclofenac (50 mg TID), and etoricoxib (30 mg OD), with and without omeprazole co-therapy (20 mg OD). These NSAIDs were chosen as there are enough data from clinical studies to allow reliable comparisons. Furthermore, these are the most commonly used agents for treating OA in Saudi Arabia [17]. Paracetamol was not included in the analysis as it is considered to have inferior efficacy compared with NSAIDs [18] and, thus, rarely used for managing pain in Saudi OA patients.

### Model design

**Fig. 1:**
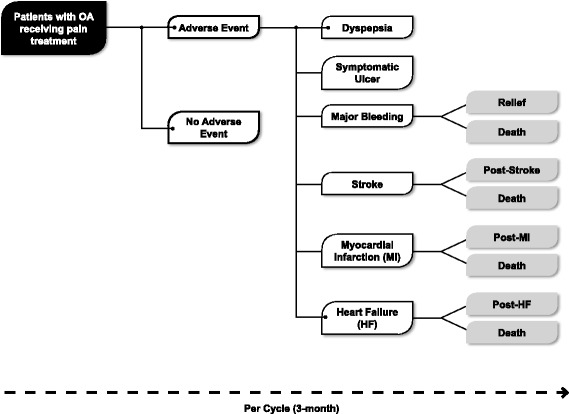
Simplified version of the cost-effectiveness model structure. OA, osteoarthritis

The CEA health model has been described elsewhere [11]. As such, the NICE health economic model developed in 2008 and updated by Brereton *et al.* with relative risk data for adverse events from the CONDOR trial was adapted for this CEA [19]. It is in the form of a Markov model with a 3-month cycle, which assumes that each patient experiences only one GI or CV event per cycle; patients may experience multiple GI and CV events over the whole time horizon (Fig. 1). Treatment duration of 6 months was adopted in the base case version of the model. The possible adverse events considered by the model included GI symptoms, symptomatic ulcer, complicated GI events, stroke, myocardial infarction and heart failure. Patients could experience multiple events at multiple points in time. The authors had previously utilized this model to evaluate the cost-effectiveness of celecoxib versus ns-NSAIDs plus PPI for treating OA in Algeria [20].

### Patient populations

The model estimated results for patients with OA aged ≥65 years. This age group was selected based on the data suggesting a 2.96-times greater risk of developing a symptomatic or complicated GI event in subjects aged ≥65 years [21].

### Adverse events

**Fig. 2:**
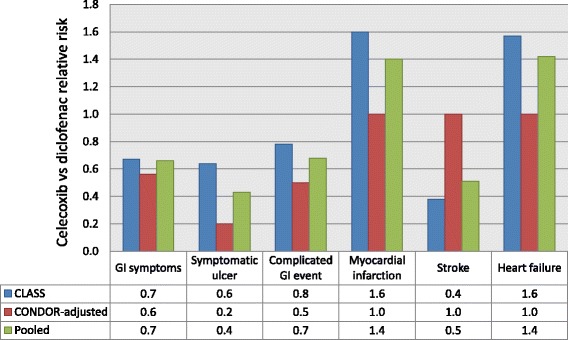
Relative risks for adverse events according to estimated treatment effects [18]. GI, gastrointestinal

Adverse event data were derived from the CLASS, MEDAL, TARGET, EDGE and CONDOR studies [6, 12–16, 19, 20]. Dose adjustments were performed in accordance with dosing regimens followed in Saudi Arabia. The overall rates of adverse events observed with different NSAIDs in these studies are shown in Table 1. Data from this table were used to perform probabilistic sensitivity analyses. Data for relative risk for each adverse event were taken from the Brereton study, which pooled data from the CLASS and CONDOR studies (Fig. 2) [19].

**Table 1:** Overall rates of adverse events observed in CONDOR, MEDAL, CLASS, EDGE and TARGET [6, 11–15]

Treatment options	Overall rates of adverse events/patient (% ± SD)					
	GI symptoms	Symptomatic ulcer	Complicated GI event	Myocardial infarction	Stroke	Heart failure
Diclofenac	21.30 (±0.9)	0.14 (±0.02)	0.07 (±0.01)	0.09 (±0.01)	0.06 (±0.01)	0.02 (±0.01)
Ibuprofen	12.72 (±0.54)	0.20 (±0.09)	0.08 ((±0.04)	0.15 (±0.11)	0.06 (±0.04)	0.09 (±0.12)
Celecoxib	12.45 (±0.46)	0.09 (±0.04)	0.05 (±0.03)	0.15 (±0.10)	0.02 (±0.02)	0.04 (±0.06)
Etoricoxib	10.40 (±0.43)	0.14 (±0.02)	0.05 (±0.03)	0.10 (±0.01)	0.06 (±0.01)	0.02 (±0.01)

GI, gastrointestinal; SD, standard deviation

### Costs

The analysis included costs incurred by patients for drug acquisition, treatment of adverse events and physician visits. The costs of treating adverse events included those associated with hospitalization, outpatient procedures and consultations, and co-prescription of PPIs (Table 2). These were estimated from data collected in July 2012 from seven private hospitals in Jeddah, Saudi Arabia. Daily treatment costs of drugs were obtained from the Saudi Ministry of Health drug list 2013 (Table 3) [22].

**Table 2:** Costs of managing specific adverse events in selected hospitals in Jeddah, Saudi Arabia

Cost of management (US $)	International Medical Center	Abu Zenada Hospital	Al Hayat Hospital	Khalid Idris Center	Al Jedaani Hospital
Dyspepsia	53.33	53.33	80.00	533.33	53.33
Symptomatic ulcer	800.00	346.67	346.67	666.67	320.00
Complicated GI event	2,680.53	1,066.67	213.33	800.00	400.00
Myocardial infarction	1,200.00	1,866.67	426.67	1,066.67	2,000.00
Stroke	2,133.33	1,866.67	426.67	1,066.67	2,000.00
Heart failure	2,133.33	1,733.33	426.67	1,066.67	2,000.00

*1 US$ = 3.75 Saudi Riyal in 2013.

Patients with dyspepsia or symptomatic ulcer were assumed to need outpatient management only.

Hospitalization costs were estimated for patients with a complicated GI event, myocardial infarction, stroke and heart failure.

Costs for managing specific adverse events were not available for United Doctors Hospital and Hai Al Jameaa Hospital.

**Table 3:** Daily costs of NSAIDs in Saudi Arabia [21]

Drug and dose	Cost/day (US$)*
Diclofenac 50 mg TID	1.16
Celecoxib 200 mg OD	0.83
Ibuprofen 400 mg TID	0.49
Omeprazole 20 mg OD	1.23
Etoricoxib 60 mg OD	1.63

NSAIDs, non-steroidal anti-inflammatory drugs

*1 US$ = 3.75 Saudi Riyal in 2013

It was assumed that patients made one consultation per adverse event, with patients with dyspepsia or symptomatic ulcer needing outpatient management only. Hospitalization costs were estimated in patients with complicated GI event, myocardial infarction, stroke and heart failure; the duration of hospitalization was based on the type and severity of the adverse event.

Costs were estimated for treatment over a period of 6 months, 2 years and 5 years. All daily cost data were expressed in 2013 prices and converted to US$ using an average 2013 exchange rate (1 US$ = 3.75 Saudi Riyal). A discount rate of 3 % was used for costs and benefits, in line with the WHO guide for conducting a CEA [23].

### QALY data

The utility scores were taken from the EQ-5D and Western Ontario and McMaster Universities Osteoarthritis Index (WOMAC) scores used in the NICE economic model [7]. EQ-5D is a standardized instrument used to measure health outcomes while WOMAC is a 24-item instrument validated for assessing pain, stiffness, and physical function in OA patients, widely used for evaluating outcomes in OA clinical trials. There was no evidence to suggest any correlation between different drug doses and drug efficacy. Thus, for this study, equal utility weights were assumed for ns-NSAIDs and COX-2 inhibitors in patients who did not experience adverse events. Utility weights for adverse events were based on data from the literature [24, 25].

### Cost-effectiveness analyses

The expected costs and QALY gains associated with eight treatment options were calculated for treatment administered over 6 months, 2 years and 5 years using deterministic analysis. Incremental cost effectiveness ratios (ICERs), defined as the additional cost per patient achieving a unit of effectiveness compared with the next less costly, non-dominated option, were calculated for each treatment duration.

### Sensitivity analyses

To assess the robustness of the model, probabilistic sensitivity analysis was performed using Monte Carlo simulations by generating 10,000 second-order iterations of the analysis. For each iteration, a different set of values was used for the parameters selected for the analysis. The 10,000 iterations generated costs and QALY results which were used to construct a cost-effectiveness acceptability curve (CEAC) that summarized the evidence in favour of a treatment arm versus comparators, and demonstrated the confidence with ICERs obtained over a range of thresholds of acceptability.

## Results

### Base case analysis

Base case analysis showed that the estimated QALY gain per patient was highest with celecoxib plus PPI (0.40) followed by etoricoxib plus PPI (0.38) and celecoxib alone (0.37) versus all comparators. The expected cost of treatment per patient was lowest with ibuprofen plus PPI (US$ 1,314.50), followed by celecoxib plus PPI (US$ 1,422.80) and celecoxib (US$ 1,543.50) (Table 4).

**Table 4:** Cost effectiveness analysis over 6-month treatment duration: Base case analysis

Therapy	Cost/patient (US$)	QALYs gained/patient	ICER (ΔC/ΔE)	Comparator
Ibuprofen + PPI	1,314.50	0.34	3,866.18	No treatment
Celecoxib + PPI	1,422.80	0.40	1,805.00	Ibuprofen + PPI
Celecoxib*	1,543.50	0.37	Dominated	-
Diclofenac + PPI*	1,565.30	0.35	Dominated	-
Ibuprofen*	1,608.20	0.28	Dominated	-
Diclofenac*	1,695.00	0.31	Dominated	-
Etoricoxib + PPI	1,749.00	0.38	Dominated	-
Etoricoxib	1,839.10	0.36	Dominated	-

*Simple dominance: Another option is less expensive and more effective

ICER, incremental cost-effectiveness ratio; PPI, proton-pump inhibitor; QALY, quality-adjusted life year

ICER: additional cost per patient achieving a unit of effectiveness compared with the next less costly, non-dominated option

The most cost effective treatment over a 6-month period was celecoxib plus PPI, with an incremental cost of US$ 108.30 and an incremental QALY gain of 0.06 resulting in an ICER of US$ 1,805.00 versus ibuprofen plus PPI (Table 4). When celecoxib plus PPI was excluded from the base case analysis, celecoxib alone was the most cost effective intervention, with an incremental cost of US$ 229.00 and an incremental QALY gain of 0.03 giving an ICER of US$ 7,633.33 versus ibuprofen plus PPI (data not shown).

The ICERs for both celecoxib alone and celecoxib plus PPI are <1 gross domestic product (GDP) per capita in Saudi Arabia in 2013 (US$ 25,961) [26], and thus considered very cost effective by the WHO-established Commission on Macroeconomics and Health [27]. Thus, celecoxib alone and celecoxib plus PPI are very cost effective treatments for Saudi Arabian OA subjects aged ≥65 years.

The other treatment options were either more expensive or less effective and, therefore, excluded by simple dominance.

### Sensitivity analyses

Analyses over 2- and 5-year treatment durations found that celecoxib plus PPI and celecoxib alone were consistently associated with higher incremental QALY gain per patient and decreasing ICERs versus comparators. Moreover, ICERs associated with celecoxib plus PPI remained lower than those of comparators across all treatment durations, making it the most cost-effective intervention for medium- and long-term use in elderly OA patients in Saudi Arabia (Tables 4, 5 and 6).

**Table 5:** Cost effectiveness analysis over 2-year treatment duration

Therapy	Cost/patient (US$)	QALYs gained/patient	ICER (ΔC/ΔE)	Comparator
Ibuprofen + PPI	4,204.10	1.11	3,787.48	No treatment
Celecoxib + PPI	4,512.80	1.37	1,187.31	Ibuprofen + PPI
Celecoxib*	4,716.80	1.24	Dominated	-
Diclofenac + PPI*	4,891.60	1.17	Dominated	-
Ibuprofen*	5,575.10	0.85	Dominated	-
Diclofenac*	5,662.50	0.96	Dominated	-
Etoricoxib + PPI	5,892.40	1.35	Dominated	-
Etoricoxib	6,621.80	1.23	Dominated	-

*Simple dominance: Another option is less expensive and more effective

ICER, incremental cost-effectiveness ratio; PPI, proton-pump inhibitor; QALY, quality-adjusted life year

ICER: additional cost per patient achieving a unit of effectiveness compared with the next less costly, non-dominated option

**Table 6:** Cost effectiveness analysis over 5-year treatment duration

Therapy	Cost/patient (US$)	QALYs gained/patient	ICER (ΔC/ΔE)	Comparator
Celecoxib + PPI	6,705.80	2.68	2,502.16	No treatment
Ibuprofen + PPI*	7,008.80	2.31	Dominated	-
Celecoxib*	7,407.10	2.50	Dominated	-
Diclofenac + PPI*	7,930.50	2.39	Dominated	-
Diclofenac*	10,140.20	2.05	Dominated	-
Ibuprofen*	10,586.10	1.83	Dominated	-
Etoricoxib + PPI	11,574.20	2.64	Dominated	-
Etoricoxib	12,983.90	2.48	Dominated	-

*Simple dominance: Another option is less expensive and more effective

ICER, incremental cost-effectiveness ratio; PPI, proton-pump inhibitor; QALY, quality-adjusted life year

ICER: additional cost per patient achieving a unit of effectiveness compared with the next less costly, non-dominated option

When celecoxib plus PPI was excluded from the analysis, celecoxib alone was the most cost effective option for treating OA in Saudi Arabia over all treatment durations (data not shown). Over 2- and 5-year treatment durations, the increased costs per patient with celecoxib alone (US$ 4,716.80 and US$ 7,407.10, respectively) were associated with highest QALYs gained per patient versus all comparators (1.24 QALYs and 2.50 QALYs, respectively), giving ICERs of US$ 3,943.85 and US$ 2,096.32, respectively, versus ibuprofen plus PPI (data not shown). We believe this is an important finding relevant for consideration in situations where PPIs may not be available. Of note, the ICERs for long-term treatment with celecoxib plus PPI and celecoxib alone were within the prescribed 1 GDP per capita for Saudi Arabia.

The robustness of findings was further confirmed with CEACs for the base case analysis. The probability that a treatment option would be cost effective was plotted against the Y-axis and the threshold cost effectiveness (the amount of money that one is willing to spend to gain one year of life) on the X-axis. Our analysis found a significantly higher likelihood that celecoxib plus PPI and celecoxib alone would be more cost effective versus comparators once the willingness to pay is over US$ 2,000.00 (data not shown).

## Discussion

After considering adverse event data from five large trials including predominantly OA patients, this CEA found that, over a 6-month treatment duration, celecoxib is more cost-effective than ns-NSAIDs, with and without PPI co-therapy, for treating Saudi Arabian OA patients aged ≥65 years. Celecoxib plus PPI remained the most cost-effective intervention even over a 5-year treatment duration. Analyses over 6-month, 2-year and 5-year treatment durations found that, next to celecoxib plus PPI, celecoxib alone had the highest QALYs gained/patient and was the most cost effective intervention. At a cost effectiveness threshold of 1 GDP per capita in Saudi Arabia, celecoxib plus PPI, as well as celecoxib alone, were highly cost effective for medium- and long-term treatment of OA in elderly Saudi Arabian patients.

Two recent economic analyses used the same economic model and clinical data and arrived at conclusions that align with our findings. A recent CEA performed in Algeria found that celecoxib plus PPI and celecoxib alone were associated with the highest QALYs gained per patient versus ns-NSAIDs with and without PPI co-therapy over 6-month and 5-year time horizons. The authors concluded that these were the most cost effective treatments for elderly OA patients in Algeria [20]. Similarly, Brereton *et al.* also showed that celecoxib plus PPI was more cost effective than diclofenac plus PPI for treating OA in the UK [19].

Could these results not have been extrapolated to the Saudi Arabian setting? Indeed, OA management varies very minimally across countries – physicians in the Middle East follow the same international treatment guidelines as doctors in Europe, and patients will present, on average, the same responses to treatment; this is why we decided to use the NICE health economic model for this CEA, the structure of which corresponds specifically to the management of OA patients. However, it would have been inaccurate to extrapolate Algerian or UK economic model outcomes to the Saudi Arabian setting as prices, treatment costs, treatment pathways, and other variables are different. Thus, the results we present are unique to Saudi Arabia and, to our knowledge, cannot be obtained from other published sources.

The findings of this CEA are in agreement with several economic analyses that show celecoxib to be more cost effective than ns-NSAIDs given with and without gastroprotective agents to patients with OA and RA [28–33]. Two economic analyses performed in Hong Kong found celecoxib to be more cost-effective than ns-NSAIDs with or without PPI co-therapy in OA and RA subjects with high GI risk [29, 32]. According to Latimer *et al.*, co-prescription of PPI with a COX-2 inhibitor rather than with an ns-NSAID was cost effective even in young subjects who have low GI risk [7]. In another study, Zabinski *et al.* found that co-prescription of ns-NSAIDs with gastroprotective agents was cost effective in high-risk patients, and the benefits accrued by using celecoxib in moderate-to-high risk patients served to offset the high costs of using celecoxib to treat low-risk patients [33]. However, this finding may be more relevant in health systems where the cost of treatment is borne by the government rather than patients.

This study has several strengths. Unlike most studies that restricted the analysis to a treatment period of 6 months, we estimated costs and benefits over 5 years of treatment. This is very relevant in OA as it is a chronic disease for which patients need long-term NSAID treatment, resulting in cumulative risk of NSAID-induced gastropathy [30]. Notably, in this model, patients with previous events were considered to have a higher likelihood of presenting with new events. Celecoxib plus PPI was the most cost effective intervention despite considering CV adverse events such as myocardial infarction, stroke and heart failure in the analysis. This is an important consideration as the elderly population included in this study may also be suffering from comorbid CV conditions. Furthermore, they may also be on prophylactic aspirin, though we did not model the increased risk of bleeding arising from aspirin therapy in this analysis. Thus, the incremental benefits from celecoxib plus PPI may well be underestimated in the current analysis.

This analysis also has several limitations. We did not stratify the estimated costs and benefits according to the age or risk category of patients. Loyd *et al.* found that the ICERs decreased when their analysis considered the increased risk of peptic ulcer bleeding with advancing age [30]. This analysis did not consider the indirect costs of OA which are known to be significant and comparable with direct costs [34]. Adverse event data were pooled from five randomized controlled trials rather than real-life situations; however, these were large studies conducted in multiple centres across several countries, and may, therefore, be considered to reflect real-life incidence of adverse events.

Some limitations are related to the economic model used for the analysis, which may have underestimated the potential benefits of treatment. For example, the model assumed that patients were maintained on the same treatment throughout the study period, whereas, in reality, patients often switch treatments due to lack of efficacy or drug intolerance. In addition, the model assumed continuous use of a drug when intermittent NSAID treatments are also known to be effective for some OA patients [30]. Only differences in GI and CV adverse event rates were modelled, with no consideration of the costs arising from any possible differences in effectiveness of the different treatments. Of note, the differences in adverse event rates derived from the randomized controlled trials were small, introducing an element of uncertainty to the sensitivity analyses.

## Conclusions

After considering new adverse risks, this CEA found that celecoxib plus PPI and celecoxib alone are very cost effective options for Saudi Arabian OA patients aged ≥65 years. Celecoxib plus PPI was the most dominant treatment over 6-month and 5-year periods, making it the most cost-effective option for medium- and long-term management of elderly OA patients in Saudi Arabia.

## Abbreviations

CEA: Cost Effectiveness Analysis
CEAC: Cost Effectiveness Acceptability Curve
COX-2 inhibitors: Cyclooxygenase-2 inhibitors
CV: Cardiovascular
GI: Gastrointestinal
ICER: Incremental Cost Effectiveness Ratio
NICE: National Institute for Health and Care Excellence
ns-NSAIDs: non-selective Non-Steroidal Anti-Inflammatory Drugs
NSAIDs: Non-Steroidal Anti-Inflammatory Drugs
OA: Osteoarthritis
PPI: Proton-Pump Inhibitors
QALYs: Quality-Adjusted Life Years
WHO: World Health Organization
WOMAC: Western Ontario and McMaster Universities Osteoarthritis Index
